# Mechanism Underlying Green Discolouration of Myoglobin Induced by Atmospheric Pressure Plasma

**DOI:** 10.1038/s41598-018-28096-4

**Published:** 2018-06-28

**Authors:** Hae In Yong, Mookyoung Han, Hyun-Joo Kim, Jeong-Yong Suh, Cheorun Jo

**Affiliations:** 1 Department of Agricultural Biotechnology, Center for Food and Bioconvergence, and Research Institute of Agriculture and Life Science, Seoul National University, Seoul, 08826 South Korea; 2 Crop Post-Harvest Technology Division, Department of Central Area Crop Science, National Institute of Crop Science, RDA, Suwon, 16613 Republic of Korea; 3 Institute of Green Bio Science and Technology, Seoul National University, Pyeongchang, 25354 Republic of Korea

## Abstract

In this study, we elucidated the mechanism underlying atmospheric pressure plasma (APP)-induced green discolouration of myoglobin. Green-coloured pigments are produced upon conversion of myoglobin into sulphmyoglobin, choleglobin, verdoheme, nitrihemin, or nitrimyoglobin. We exposed myoglobin dissolved in phosphate buffer to APP for 20 min and found a decrease in a^*^ value (+redness/−greenness) and increase in b^*^ value (+yellowness/−blueness) (*P* < 0.05). In the ultraviolet absorption spectrum, myoglobin treated with APP for 20 min showed absorption peaks at 503 and 630 nm, a spectrum different from that of sulphmyoglobin or choleglobin. The secondary structure and molecular weight of myoglobin were unaffected by APP treatment, excluding the possibility of verdoheme or nitrihemin formation. After APP treatment, nitrite was produced in myoglobin solution that provided a positive environment for nitrimyoglobin formation. However, the addition of 0.5% sodium dithionite, a strong reducing agent, to myoglobin solution resulted in the formation of deoxymyoglobin, which was subsequently converted to nitrosomyoglobin upon APP treatment to yield a desirable red colour. Thus, APP-induced green colouration in myoglobin solution is associated with nitrimyoglobin formation. The addition of the antioxidant resulted in the production of red colour in myoglobin solution after APP treatment owing to nitrosomyoglobin formation.

## Introduction

The emergence of food-borne illnesses has led to the development of novel non-thermal decontamination technologies such as the atmospheric pressure plasma (APP) technology. Plasma, produced by electric gas discharges, comprises various reactive species, free radicals, electrons, and ions^1,2^. To date, bactericidal and virucidal effects of APP on meat are well documented. In previous study, *Escherichia coli* population on the raw chicken was reduced by 1.5 to 2.0 Log CFU/g after APP jet exposure for 10 min^3^. Additionally, the numbers of *E. coli* O157:H7, *Listeria monocytogenes*, and *Salmonella* Typhimurium were significantly reduced in both pork and beef with increase of APP treatment time^4^. Following 10 min of APP treatment, total aerobic bacterial count in chicken samples were also reduced by 3.36 Log CFU/g^5^. Bae *et al*. reported approximately 2 and 1.5 Log PFU/mL reductions in murine-norovirus and hepatitis A virus levels, respectively, in raw meat after APP jet treatment for 20 min^6^.

However, some studies reported that APP treatment cause minor deterioration in meat quality, especially the meat colour^1,7^. The exposure to dielectric barrier discharge plasma using atmospheric air significantly lowered a^*^ values of raw chicken breast, pork butt, and beef loin^4,5^. The decrease in a^*^ value is characterised with a reduction in the meat redness and induction of meat greenness. The application of indirect plasma system using processed air gas to raw pork (*M. longissimus dorsi*) resulted in the greener appearance of samples^8^. Kim *et al*. reported the green discolouration of raw pork loin after plasma treatment with helium gas. In addition, the sensory score for colour was significantly lower in plasma-treated raw pork as compared with the untreated control sample^9^.

Meat colour is the most important quality attribute because it influences the first impression and purchasing decision of the consumers. Consumers generally use the meat colour as an indicator of freshness or wholesomeness^10^. Around 15% of retail beef are discounted in price following their discolouration, resulting in an annual revenue loss of 1 billion dollars in the US^11^. Therefore, it is important to maintain the fresh meat colour after APP treatment to improve the safety and extend the shelf-life of the meat^7^. Thus, green discolouration of meat caused by APP treatment should be investigated and controlled.

The factor associated with the meat colour is myoglobin, a monomeric protein composed of a globin protein and haeme group. In the haeme group, an iron atom is centrally located in a porphyrin ring to form six bonds. Four of these bonds coordinate with pyrrole nitrogen atoms in the porphyrin ring and one is associated with the proximal histidine of globin protein. In addition, the sixth site of iron is available to bind electronegative atoms of various ligands^12,13^. Myoglobin colour is determined by the type of ligands (O_2_, H_2_O, NO, CO, etc.) bound to the iron, the oxidation state of iron (ferrous or ferric), and the integrity of porphyrin ring or globin protein^14^. Primary forms of myoglobin found in raw meat are deoxymyoglobin (no ligand, ferrous iron, native globin protein), oxymyoglobin (ligand-O_2_, ferrous iron, native globin protein), and metmyoglobin (ligand-H_2_O, ferric iron, native globin protein) with purplish-red, bright cherish-red, and brownish-red colour, respectively^10^.

We hypothesised that APP treatment affects myoglobin in raw meat, resulting in meat discolouration. Previously, Attri *et al*. reported modification in the myoglobin structure after APP treatment; however, the purpose of the study was not elucidation of meat discolouration induced by APP but identification of biocompatibility of APP in biomedical engineering^15^. A fundamental explanation of the interaction between APP and myoglobin and the consequent colour change is still insufficient. In this study, we elucidate the mechanism underlying APP-induced green discolouration of myoglobin (Experiment I) and demonstrate a control measure (Experiment II).

## Methods

### Experiment I: Elucidation of green discolouration of myoglobin

Horse skeletal muscle myoglobin was purchased from Sigma Chemical Co. (St. Louis, MO, USA) and used without further purification in the form of metmyoglobin, which has ferric iron and no bound oxygen. In addition, it possesses no disulphide bridges or free -SH groups in globin protein. Myoglobin (60 mM) was dissolved in distilled water (DW) and 0.4 M sodium phosphate buffer (pH 6.8). Colour and pH changes of myoglobin in DW and phosphate buffer were measured after APP treatment. UV absorption spectra, secondary protein structure, molecular weight, and concentration of nitrite and nitrate were evaluated for the myoglobin solution in phosphate buffer.

### Experiment II: Control of green discolouration of myoglobin

Horse skeletal muscle myoglobin was dissolved in phosphate buffer and treated with 0.1% and 0.5% sodium dithionite. The samples were analysed for their colour and ultraviolet (UV) absorption spectra.

### Treatment with APP

An encapsulated APP apparatus fabricated with a rectangular plastic container (13.7 × 10.4 × 5.3 cm^3^) used a dielectric barrier discharge (DBD). DBD actuator was made of copper electrodes and a dielectric polytetrafluoroethylene sheet, and this actuator was attached to inner walls of the container. At the bottom and centre of the container, myoglobin solution (20 mL) was placed in a glass dish. Distance between the surface of the myoglobin solution and the ground electrode was 1.2 cm (Fig. S1). APP treatment was performed as previously described^2^. Atmospheric air in its natural state was used as a carrier gas without using other input gases and gas flow. A sine-waveform voltage at 15 kHz was applied to one electrode while the other electrode was grounded and the plasma was generated at 250 W input power inside the container.

### Colour value and pH

The colour of the myoglobin solution was measured using a quartz cell with a colorimeter (CR-5, Minolta Camera Co., Osaka, Japan). The instrument was calibrated with a standard black-plate and DW before analysis. The colour values were expressed as *L*^∗^ (+brightness, −darkness), *a*^∗^ (+redness, −greenness), and *b*^∗^ (+yellowness, −blueness) values. A more appropriate measure of colour was obtained from the total colour difference$\,({\rm{\Delta }}E=\sqrt{{({\rm{\Delta }}{L}^{\ast })}^{2}+{({\rm{\Delta }}{a}^{\ast })}^{2}+{({\rm{\Delta }}{b}^{\ast })}^{2}})$and chroma $(\sqrt{{({a}^{\ast })}^{2}+{({b}^{\ast })}^{2}})$ that were calculated from L^*^, a^*^, and b^*^ values. Chroma (saturation index) refers to vivid or dull colour and is proportional to the colour intensity.

The pH of each sample was measured using a pH meter (SevenGo, Mettler-Toledo International Inc., Schwerzenbach, Switzerland).

### Ultraviolet absorbance spectrum

Absorption scans of the myoglobin solution were conducted from 380 to 600 nm at 1 nm increment using a Model X-ma 3100 spectrophotometer (Human Co., Ltd., Seoul, Korea).

### Circular dichroism (CD) and electrospray ionisation-mass spectrometry (ESI-MS) spectra

Chirascan plus spectrophotometer (Applied Photophysics, Leatherhead, UK) was used for CD measurements. The instrument was set at a scan rate of 50 nm/min, a bandwidth of 0.5 nm, and an integration time of 1 s.

The samples were analysed on a Triple TOF 5600 Q-TOF LC/MS/MS system (Ab Sciex, Redwood City, CA, USA) using an Ultimate 3000 RSLC HPLC system (Thermo Fisher Scientific Inc., Waltham, MA, USA) including a degasser, an auto-sampler, a diode array detector, and a binary pump. LC separation was performed on a column (Zorbax, 3.5 um, 300SB-C8, 2.1 × 50 mm, PN865750-906, Agilent Technologies Inc., Santa Clara, USA) with a mobile phase A (0.1% formic acid in water) and mobile phase B (0.1% formic acid in acetonitrile). The flow rate was 0.25 mL/min. The auto-sampler was set at 4 °C and the injection volume was 1–5 μL. Mass spectra were acquired under positive electrospray ionisation (ESI) with an ion spray voltage of 5500 V. The source temperature was 500 °C. The pressures of curtain gas, ion source gas 1, and ion source gas 2 were 50, 50, and 25 psi, respectively. Two full-scan mass spectra were acquired over an m/z range of 350–1800 on MS mode. The data were collected using Analyst TF 1.7 software and analysed using PeakView 2.2., Bio Tool Kit 2.2.0.

### Nitrite and nitrate concentrations

Myoglobin solutions were filtered through a 0.2-μm polyvinylidene fluoride syringe filter (Whatman Inc., Maidstone, Kent, UK) and diluted with distilled water (1:200, v/v). Nitrite and nitrate concentrations were measured using an ion-chromatograph (Dionex ICS-3000; Dionex Corporation, Sunnyvale, USA) equipped with a dual eluent generator system, dual chromatography compartments with dual suppressed conductivity detectors, and dual gradient pumps. Samples were analysed using a guard column, AG 20 (50 × 2.0 mm inner diameter, Dionex Corporation, Sunnyvale, USA) coupled with an IonPac AS20 (250 × 4.0 mm inner diameter, Dionex Corporation, Sunnyvale, USA) analytical column. The flow rate was 1 mL/min. Suppression was achieved using an ASRS URTRA II (4 mm) self-regenerating suppressor, and the injection volume was 25 µL. Analyses were performed with a gradient elution mode, starting with 15 mM of potassium hydroxide for 8 min, followed by 40 mM from 8–18 min and 15 mM from 19–20 min.

### Statistical analysis

Each set of data represents the mean of three replicates. Statistical analysis was performed by one-way analysis of variance (ANOVA) with a completely randomised design using the procedure of General Linear Model. Significant differences between mean values were identified using the Tukey’s multiple comparison test in SAS software 9.4. (SAS Institute Inc., Cary, NC, USA) at a significance level of *P* < 0.05.

## Results and Discussion

### Experiment I

#### Colour and pH

**Figure 1:**
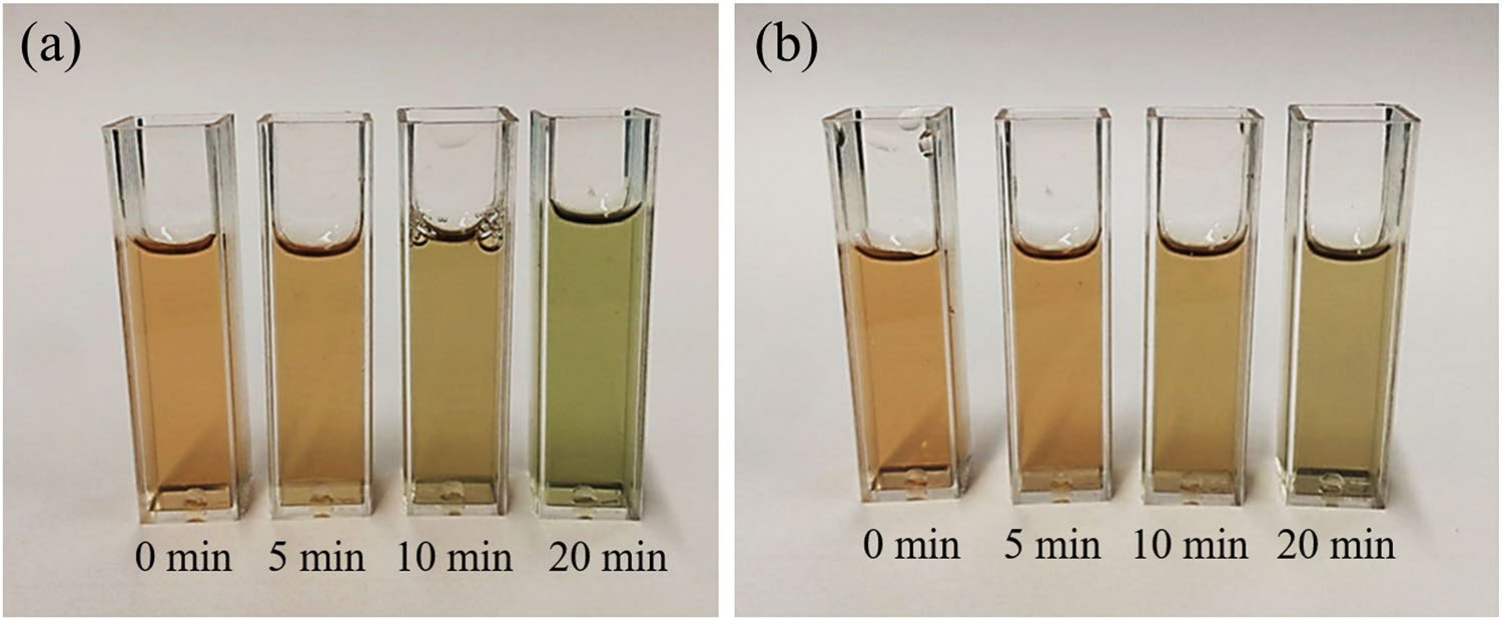
Visual appearance of myoglobin in phosphate buffer (**a**) and DW (**b**) after exposure to atmospheric pressure plasma for 0, 5, 10, and 20 min.

We observed a significant decrease in L^*^ and a^*^ values of myoglobin in both phosphate buffer and DW with an increasing APP treatment time (Table 1). While the values of b^*^ and chroma for myoglobin dissolved in phosphate buffer decreased with an increasing APP treatment time (*P* < 0.05), those for myoglobin dissolved in DW decreased only up to 10 min of APP treatment and then slightly increased after 20 min of APP treatment (*P* < 0.05). The value of Δ*E* was lower for myoglobin in phosphate buffer than that in DW. These results indicate that myoglobin in phosphate buffer and DW showed yellowish green and blush green colour, respectively, after 20 min of APP treatment. In addition, the green discolouration of myoglobin following APP treatment was more evident in DW than in phosphate buffer. Visual appearances of myoglobin solutions are shown in Fig. 1.

According to Renerre and Brewer, the porphyrin ring of myoglobin is denatured at pH below 5.0, resulting in the production of green pigments^12,16^. We observed that myoglobin in DW turned green following 10 and 20 min APP treatment, and the pH was below 5.0. (Table 1). It is well known that APP exposure reduces the pH value of liquids, owing to the generation of acidogenic molecules (e.g., nitric oxide [NO], nitrogen dioxide [NO_2_], nitrous acid [HNO_2_], and nitric acid [HNO_3_])^17^. However, green colouration was also observed from myoglobin dissolved in phosphate buffer after 20-min APP treatment, although the pH value of the sample was above 5.0 (Table 1). As raw meat exhibits buffering capacity similar to that of the phosphate buffer, it is difficult to reduce the pH value of raw meat by APP treatment^18^. Previous studies have reported that pH values of APP-treated raw pork were above 5.30 when the samples showed greener appearance^8,9^. Therefore, green discolouration of raw meat with APP treatment is associated with factors other than low pH. In the following experiments, myoglobin was dissolved in phosphate buffer.

**Table 1:** Colour and pH value of myoglobin dissolved in phosphate buffer and distilled water following exposure of atmospheric pressure plasma.

Properties	Treatment time (min)				SEM^1^
	0	5	10	20	
**Myoglobin in phosphate buffer**					
*L* ^*^	79.92^a^	79.60^a^	79.10^b^	78.48^c^	0.091
*a* ^*^	9.50^a^	7.87^b^	6.58^b^	4.86^c^	0.343
*b* ^*^	30.41^a^	29.83^b^	29.77^b^	29.58^c^	0.034
Chroma	31.86^a^	30.85^b^	30.49^c^	29.99^d^	0.033
Δ*E*	0.00^c^	1.76^b^	3.10^b^	4.94^a^	0.332
pH	6.86^a^	6.85^a^	6.82^ab^	6.80^b^	0.010
**Myoglobin in DW**					
*L* ^*^	62.87^a^	60.32^b^	58.41^c^	57.93^d^	0.015
*a* ^*^	18.31^a^	15.83^b^	9.69^c^	0.21^d^	0.015
*b* ^*^	42.40^a^	38.86^c^	36.33^d^	39.44^b^	0.016
Chroma	46.19^a^	41.96^b^	37.60^d^	39.44^c^	0.018
Δ*E*	0.00^d^	5.03^c^	11.46^b^	19.00^a^	0.019
pH	6.43^a^	5.50^b^	4.21^c^	3.39^d^	0.074

^1^Standard error of the mean (n = 12).

^a,b,c,d^Values with different letters within the same row differ significantly (*P* < 0.05).

In general, myoglobin appears green upon its conversion to sulphmyoglobin, choleglobin, verdoheme, nitrimyoglobin, and nitrihemin under specific conditions. Sulphmyoglobin is produced in response to the reaction between hydrogen sulphide (H_2_S) and deoxymyoglobin in vacuum-packaged raw meat. However, sulphmyoglobin becomes oxygenated to metsulphmyoglobin once the vacuum-packaged meat is opened, giving it a red appearance. Sulphmyoglobin is found only in meat with low oxygen concentration ranging from 1% to 2%^13,14^. In the present study, myoglobin solution was exposed to APP under aerobic condition and hence, the possibility of sulphmyoglobin formation was low. Formation of choleglobin is induced following the reaction of myoglobin with highly oxidising agents such as hydrogen peroxide. Choleglobin has a hydroperoxide (-OOH) group attached at the sixth coordination site of ferric or ferrous iron. In addition, further oxidation of choleglobin result in the opening of the porphyrin ring (iron remains) to yield green verdoheme^19,20^. Nitrimyoglobin is formed upon the exposure of metmyoglobin to excess nitrite and HNO_2_ at pH values below 7.0. Nitrimyoglobin is one of the concerns in meat industry, as it produces green discolouration in cured meat product, a process called as “nitrite-burn”^21,22^. Upon its heating and reaction with HNO_2_, nitrimyoglobin is converted to nitrihemin, one of the green pigments^10^. Unlike sulphmyoglobin, choleglobin, and nitrimyoglobin, the globin protein is absent in verdoheme and nitrihemin^20^.

We hypothesised that the green discolouration of myoglobin by APP treatment is caused by one or more of the above-mentioned green pigments.

#### Ultraviolet absorption spectra

**Figure 2:**
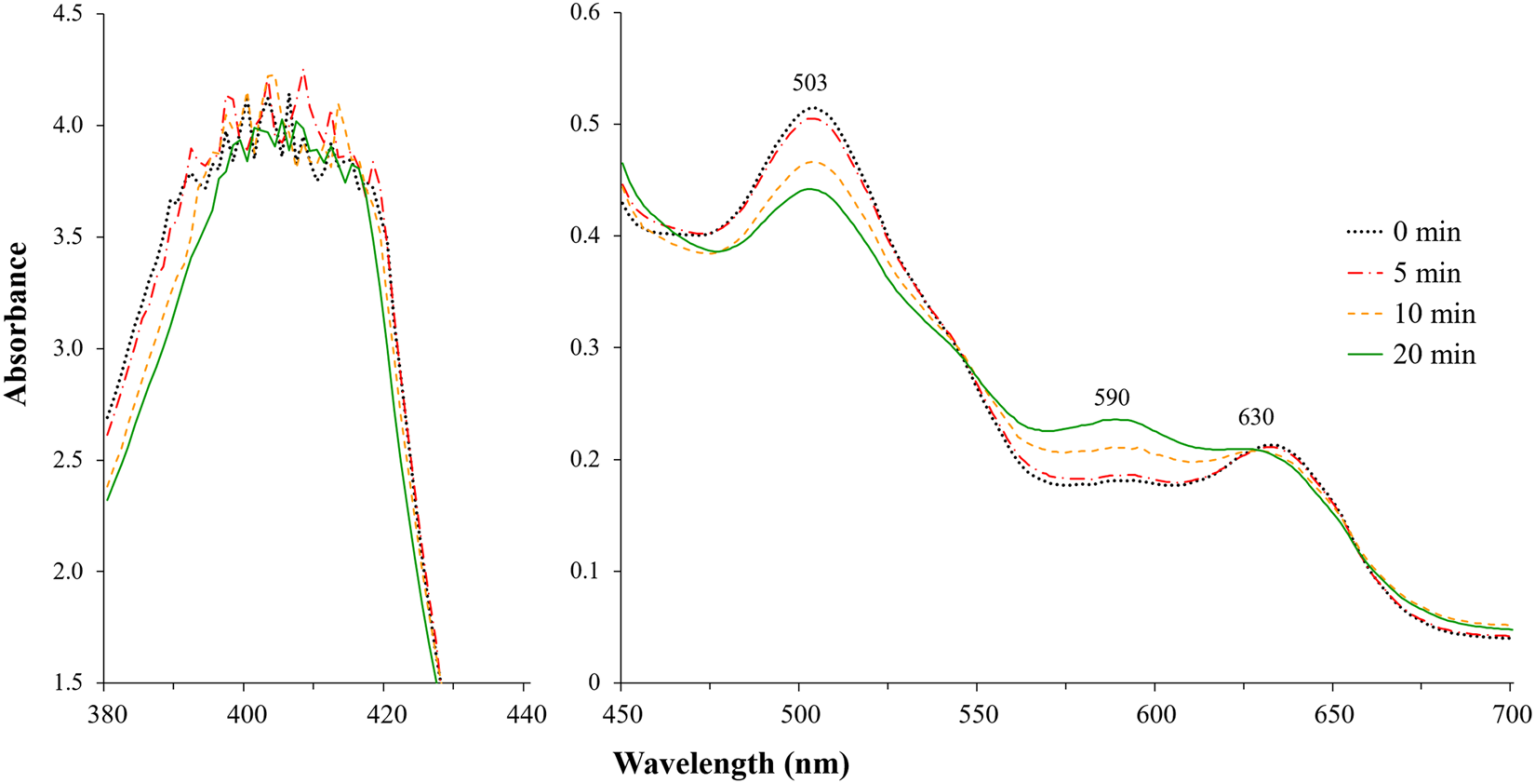
UV absorption spectrum of myoglobin in phosphate buffer after exposure to atmospheric pressure plasma.

The analysis of UV absorption spectrum of myoglobin solution revealed a decrease in the absorption peaks at 503 and 630 nm with an increase in the peak at 590 nm in response to an increasing APP treatment time (*P* < 0.05, Fig. 2). The absorption spectra of haeme pigments, including myoglobin, vary depending on the physical and chemical state of the pigments^23^. Sulphmyoglobin and choleglobin have their typical spectra with absorption maximum at 615 and 628 nm, respectively^12,13,24^; however, these maximum peaks were absent in myoglobin solution subjected to APP treatment for 20 min. From these results, it could be concluded that both sulphmyoglobin and choleglobin were absent.

We observed a typical spectrum of the haeme group from UV absorption spectrum of myoglobin solution, with maximum absorbance at around 409 nm, termed as “Soret band.” Depending on the chemical state of iron (ferric or ferrous) located in the porphyrin ring, Soret band of the haeme group is shifted to a longer or shorter wavelength^12,25^. In Fig. 2, Soret peak intensity of myoglobin solution failed to shift to other wavelengths after APP treatment but slightly decrease with an increasing APP treatment time. The decrease in Soret band indicates the partial denaturation of the porphyrin ring in haeme group^26,27^. To investigate whether APP affects the porphyrin ring, protoporphyrin IX (purchased in Sigma Chemical Co., USA) was dissolved in 0.4 M sodium phosphate buffer (pH 6.8) and exposed to APP for 0, 5, 10, and 20 min (Table S1). Similar to the discolouration of myoglobin in phosphate buffer (Table 1), L^*^, a^*^, b^*^, and chroma values decreased and Δ*E* value increased for protoporphyrin IX solution with an increasing APP treatment time. Thus, green discolouration was induced in protoporphyrin IX solution following APP treatment. Therefore, the occurrence of APP-induced green discolouration of myoglobin is related to the changes in the porphyrin ring structure.

#### Circular dichroism and ESI-MS spectra

**Figure 3:**
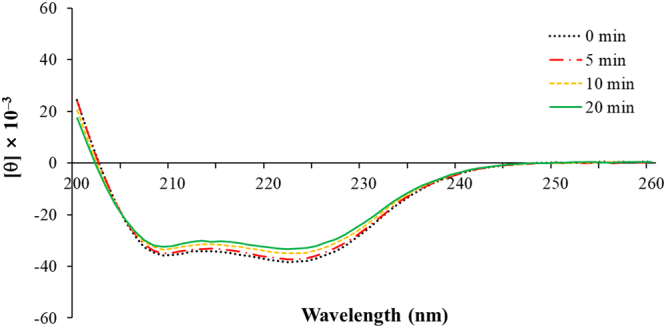
Circular dichroism spectrum of myoglobin in phosphate buffer after exposure to atmospheric pressure plasma.

Horse myoglobin has a single chain of 153 amino acids with a globin fold that contains eight α-helical segments (A–H) enwrapping the haeme group^12^. As shown in Fig. 3, the secondary protein structure of myoglobin comprised 64.2% α-helix, 0.6% β-sheet, 11.9% β-turn, and 17.0% random coil. These structures were unaffected by APP exposure for up to 20 min. Attri *et al*. reported an increase in the amount of α-helix and a decrease in β-sheet in myoglobin after its exposure to air plasma^15^. On the contrary, a decrease in α-helical structure and an increase in β-sheet structure were observed in myoglobin treated with nitrogen plasma and argon plasma, respectively^15^. Thus, the changes in the secondary structure of myoglobin are affected by the gas applied to the plasma discharge.

We also failed to observe any alteration in the molecular weight of myoglobin (16,951 Da) after APP treatment for up to 20 min, which indicates the absence of any degradation or aggregation of myoglobin by APP (Fig. S2). Verdoheme and nitrihemin, the green pigments, does not have globin protein^20^. If the globin protein was detached from the heme-group by APP exposure, the molecular weight of myoglobin must be decreased. Thus, verdoheme and nitrihemin had no contribution to the green discolouration of myoglobin solution following APP treatment.

#### Nitrite and nitrate concentrations

**Figure 4:**
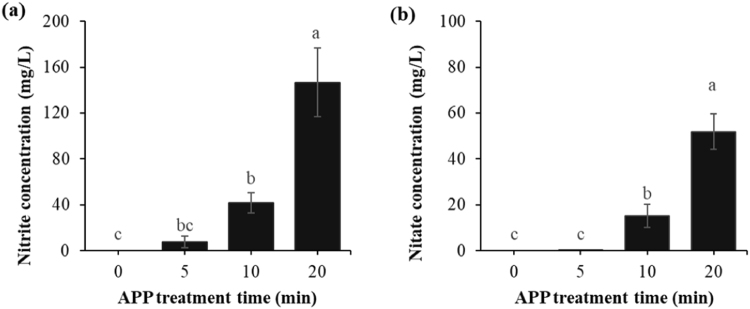
Nitrite (**a**) and nitrate (**b**) concentrations of myoglobin in phosphate buffer after exposure to atmospheric pressure plasma. a-c Different letters indicate significant differences (*P* < 0.05).

In order to investigate the possibility of the formation of nitrimyoglobin in response to APP treatment, nitrite and nitrate concentrations were measured in the myoglobin solution. When APP treatment time increased more than 10 min, nitrite and nitrate concentrations of myoglobin solution significantly increased (*P* < 0.05, Fig. 4). The concentrations of nitrites and nitrates in myoglobin solution treated with APP for 20 min were 146.72 and 51.87 mg/mL, respectively.

In general, APP discharged with atmospheric air contains various reactive nitrogen species, including NO, which diffuses and dissolves in liquids. Dissolved NO react with oxygen (O_2_) present in liquids to generate NO_2_. The reaction between NO_2_ and water molecule results in the formation of HNO_2_ and HNO_3_, as described in equations 1–3^28,29^.

1 $${\rm{NO}}+{{\rm{O}}}_{2}\to 2{{\rm{NO}}}_{2}$$

2 $${\rm{NO}}+{{\rm{NO}}}_{2}+{{\rm{H}}}_{2}{\rm{O}}\to 2{{\rm{HNO}}}_{2}$$

3 $${{\rm{NO}}}_{2}+{{\rm{NO}}}_{2}+{{\rm{H}}}_{2}{\rm{O}}\to {{\rm{HNO}}}_{2}+{{\rm{HNO}}}_{3}$$

4 $${{\rm{HNO}}}_{2}\leftrightarrow {{\rm{NO}}}_{2}^{-}+{{\rm{H}}}^{+}$$

5 $$3{{\rm{NO}}}_{2}^{-}+{{\rm{H}}}^{+}+{{\rm{H}}}_{2}{{\rm{O}}}_{2}\to {{\rm{NO}}}_{3}^{-}+2{\rm{NO}}+{{\rm{H}}}_{3}{{\rm{O}}}^{+}$$

As shown in Equation 4, HNO_2_ (p*Ka* = 2.8–3.2) release hydrogen ions, resulting in the acidification of liquid. Nitrite is subsequently oxidised into nitrate under acidic conditions (Equation 5). However, nitrite in an alkaline-buffered condition is not oxidised to nitrate, thus maintaining its concentration^28,30^. Braida and Ong reported that the oxidation rate of nitrite to nitrate is dependent on the pH of the liquid and is remarkably lower at pH 5.80 than at pH 2.85^31^. As the pH value of myoglobin in phosphate buffer was 6.80 after APP treatment for 20 min (Table 1), the concentration of nitrite was higher than that of nitrate (Fig. 4).

Generally, chemical species in liquid phase comply with the usual solution chemistry laws such as electron exchange reactions (oxidation/reduction), Bronsted acidity (proton exchange), and Lewis acidity (ligand exchange or complex formation). In previous studies about APP treated solutions, Lewis acid-base reaction was not yet well reported unlike others chemistry laws^32^. However, Brisset and Pawlat suggested the possibility of the Lewis acid-base reaction after APP treatment. For example, it was well known that NO could be generated in APP treated solution^32^, and the NO can forms adduct with Fe^2+^-porphyrin in axial position^33^. In this respect, the results of the present study could be due to the Lewis acid-base reaction between nitrite generated by APP and myoglobin in solution.

Actually, it was reported that the excess of nitrite addition to metmyoglobin produce metmyoglobin-nitrite (reddish-brown colour). The treatment of metmyoglobin-nitrite with an even higher concentrations of nitrite and HNO_2_ results in the formation of nitrimyoglobin (green colour)^19–22^. The high concentration of nitrite produced by APP may form metmyoglobin-nitrite from myoglobin solution used in the present study. Nitrimyoglobin can be eventually formed from metmyoglobin-nitrite, owing to the nitrite and HNO_2_ produced through Equation 4.

The structure of nitrimyoglobin revealed that the haeme group at 2-vinyl position was nitrated. Thus, the haeme group in nitrimyoglobin has nitrovinyl porphyrin such as 3-(trans-2-nitrovinyl)-2,7,12,18-tetramethyl-8-vinylporphyrin-13,17-dipropionic acid^21,34^. UV absorption spectrum of myoglobin solution revealed the denaturation of porphyrin ring by APP (Fig. 2). It might be due to nitration of porphyrin ring owing to nitrimyoglobin formation. The experimental results of the present study support the formation of nitrimyoglobin after APP treatment of myoglobin solution.

### Experiment II

#### Colour and UV absorption spectra

In order to control the green discolouration of myoglobin by APP treatment, sodium dithionite, a strong reducing agent, was added to myoglobin solution after APP treatment (Table 2). We observed that APP treatment for 20 min had no effect on L^*^, b^*^, and chroma values and a^*^ value decreased by 2.32 (*P* < 0.05) for myoglobin solution treated with 0.1% sodium dithionite. After 20 min of APP treatment, Δ*E* values were 2.86 and 4.94 for myoglobin solution with or without 0.1% sodium dithionite, respectively (Tables 1 and 2). Therefore, the addition of 0.1% sodium dithionite reduce APP-induced green discolouration of myoglobin. Myoglobin solution treated with 0.5% sodium dithionite showed no significant difference in L^*^ and b^*^ values, but a^*^, chroma, and Δ*E* values increased after 20 min of APP treatment. APP treatment of myoglobin solution supplemented with 0.5% sodium dithionite for 20 min resulted in the production of red colour instead of the green discolouration. Visual appearance of myoglobin, and myoglobin supplemented with 0.1 and 0.5% sodium dithionite following exposure to atmospheric pressure plasma for 20 min were shown in Fig. S3.

**Table 2:** Colour of myoglobin dissolved in phosphate buffer supplemented with 0.1% and 0.5% sodium dithionite following exposure to atmospheric pressure plasma.

Properties	Treatment time (min)		SEM^1^
	0	20	
**Myoglobin in the presence of 0.1% sodium dithionite**			
*L* ^*^	77.09	77.35	0.507
*a* ^*^	10.31^a^	7.99^b^	0.304
*b* ^*^	34.91	35.12	0.418
Chroma	36.40	36.02	0.360
Δ*E*	0^b^	2.86^a^	0.224
**Myoglobin in the presence of 0.5% sodium dithionite**			
*L* ^*^	73.89	72.85	0.285
*a* ^*^	4.56^b^	11.94^a^	0.237
*b* ^*^	29.32	33.43	1.312
Chroma	29.67^b^	35.52^a^	1.173
Δ*E*	0^b^	8.94^a^	0.310

^1^Standard error of the mean (n = 6).

^a,b,c,d^Values with different letters within the same row differ significantly (*P* < 0.05).

**Figure 5:**
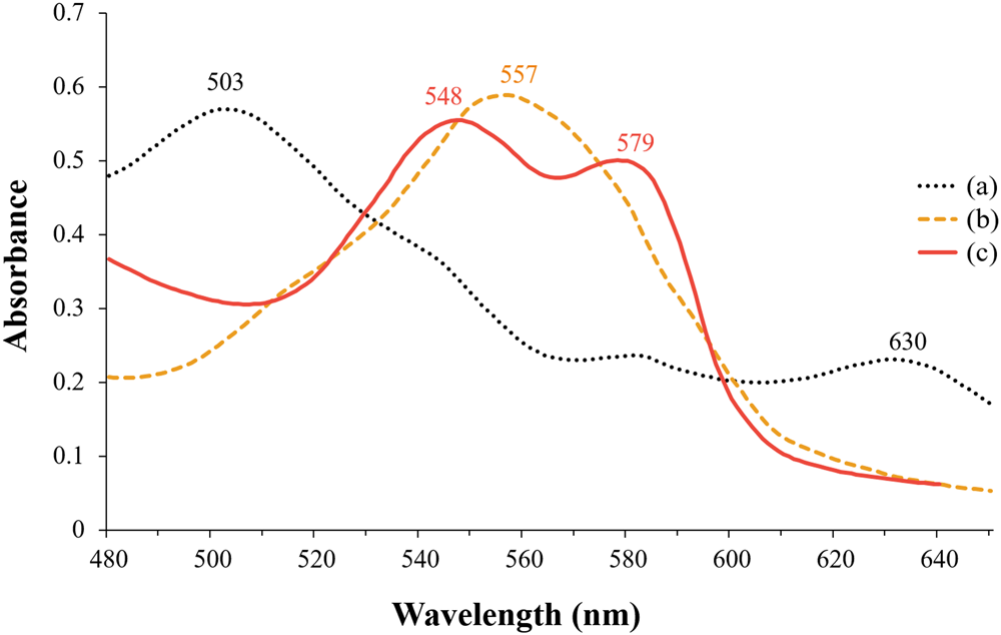
UV absorption spectra of myoglobin (**a**), myoglobin with 0.5% sodium dithionite (**b**), and myoglobin with 0.5% sodium dithionite treated with atmospheric pressure plasma (APP) for 20 min (**c**). All myoglobin samples were dissolved in phosphate buffer.

UV absorption spectra was measured to evaluate the red colouration of myoglobin solution following treatment with APP in the presence of 0.5% sodium dithionite, and it was significantly changed depending on the treatment (*P* < 0.05). Myoglobin solution used in the present study comprised metmyoglobin and had an absorption peak at 503 and 630 nm (Fig. 5(a)). Myoglobin solution treated with 0.5% sodium dithionite showed an absorption peak at 577 nm, with a typical spectrum of deoxymyoglobin (Fig. 5(b))^23,25^. Ferric iron in metmyoglobin is reduced to ferrous iron by the reducing agent, resulting in the formation of deoxymyoglobin^10,13^. The addition of 0.5% sodium dithionite to myoglobin solution followed by APP treatment for 20 min produced absorption peaks at 548 and 579 nm (Fig. 5(c)). This observation is in line with the characteristic absorption spectrum of nitrosomyoglobin (bright red colour). Nitrosomyoglobin shows higher absorption peak at 548 nm as compared to 579 nm^23^. In general, the reaction between deoxymyoglobin and NO produces nitrosomyoglobin, also known as nitrosylmyoglobin^13,25^.

**Figure 6:**
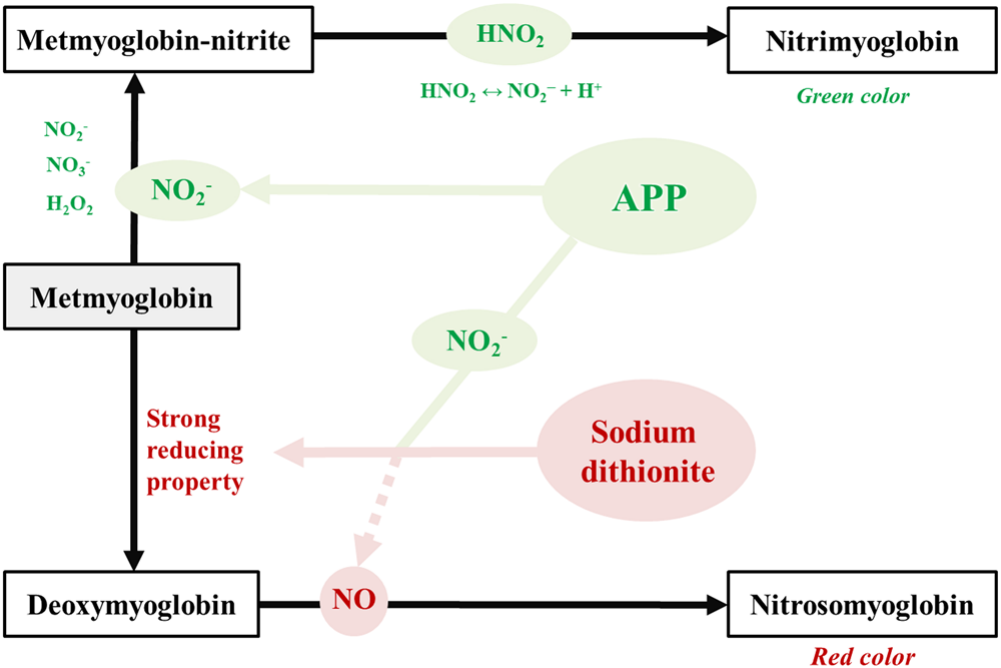
Schematic of the effect of atmospheric pressure plasma (APP) and sodium dithionite on metmyoglobin.

According to Fox Jr and Thomson, a green haeme pigment termed as nitrimyoglobin was formed upon incubation of nitrite, metmyoglobin, and hydrogen ion in the absence of ascorbic acid^35^. On the contrary, the addition of ascorbic acid to the above system resulted in the formation of nitrosomyoglobin, owing to the reducing action of ascorbic acid. These experimental results of Fox Jr and Thomson revealed the conditions involved in the formation of nitrimyoglobin and nitrosomyoglobin^35^ and are in accordance with the results of the present study. The effect of APP and sodium dithionite on metmyoglobin is illustrated in Fig. 6.

During the manufacturing of meat products, nitrite is generally added to meat to produce nitrosomyoglobin to present a desirable bright red colour to the meat. Off-coloured raw meat (green or grey) cannot meet consumer choice^13,30^. Thus, the results of the present study is useful to prevent the formation of nitrimyoglobin (greenish colour). Yong *et al*. reported that APP treatment increases redness and nitroso-haeme pigment in pork jerky in the presence of ascorbic acid^36^.

## Conclusion

The mechanism underlying APP-induced green discolouration of myoglobin was investigated and a control measure was developed. Myoglobin-derived green pigments generally involve sulphmyoglobin, choleglobin, verdoheme, and nitrihemin, none of which were responsible for the green discoloration after APP treatment in this study. Instead, nitrite generated by APP can react with myoglobin to form nitrimyoglobin which has the green colour. Reducing agent such as sodium dithionite (0.1%) prevent APP-induced green discolouration. Moreover, higher concentration (5%) of the reducing agent induce red colouration through the formation of nitrosomyoglobin.

This is the first study to reveal the mechanism underlying APP-induced green discolouration of myoglobin. Any food processing methods have their own advantages and disadvantages. The findings of this study can be applied to accelerate the application of new technology, APP, in the industry to improve food safety and processing by minimising undesirable quality changes.

## Electronic supplementary material

Supplementary information

## References

[1] Lee J (2017). Use of atmospheric pressure cold plasma for meat industry. Korean J. Food Sci. An..

[2] Yong HI (2015). Evaluation of pathogen inactivation on sliced cheese induced by encapsulated atmospheric pressure dielectric barrier discharge plasma. Food Microbiol..

[3] Yong HI (2014). Evaluation of the treatment of both sides of raw chicken breasts with an atmospheric pressure plasma jet for the inactivation of *Escherichia coli*. Foodborne Pathog. Dis..

[4] Jayasena DD (2015). Flexible thin-layer dielectric barrier discharge plasma treatment of pork butt and beef loin: Effects on pathogen inactivation and meat-quality attributes. Food Microbiol..

[5] Lee H (2016). Evaluation of the microbiological safety, quality changes, and genotoxicity of chicken breast treated with flexible thin-layer dielectric barrier discharge plasma. Food Sci. Biotechnol..

[6] Bae SC, Park SY, Choe W, Ha SD (2015). Inactivation of murine norovirus-1 and hepatitis A virus on fresh meats by atmospheric pressure plasma jets. Food Res. Int..

[7] Misra NN, Jo C (2017). Applications of cold plasma technology for microbiological-safety in meat industry. Trends Food. Sci. Technol..

[8] Fröhling A (2012). Indirect plasma treatment of fresh pork: Decontamination efficiency and effects on quality attributes. Innov. Food Sci. Emerg. Technol..

[9] Kim HJ, Yong HI, Park S, Choe W, Jo C (2013). Effects of dielectric barrier discharge plasma on pathogen inactivation and the physicochemical and sensory characteristics of pork loin. Curr. Appl. Phys..

[10] Schwartz, S., von Elbe, J. & Giusti, M. Colorants. Fennema’s food chemistry-4th ed (eds Damodaran, S., Parkin, K. &Fennema, O) pp. 571–638 (Taylor & Francis, 2008).

[11] Smith, G. C., Belk, K. E., Sofos, J. N., Tatum, J. D. & Williams, S. N. Economic implications of improved color stability in beef. Antioxidants in muscle foods: Nutritional strategies to improve quality. (eds Decker, E. A., Faustman, C. & Lopez-Bote, C. J.) 397–426 (New York: Wiley Interscience, 2000).

[12] Brewer S (2004). Irradiation effects on meat color–a review. Meat Sci..

[13] DeMan, J. M. Color. In *principles of food chemistry-3ed ed*. 239–242 (Springer US, 1999).

[14] Seideman SC, Cross HR, Smith GC, Durland PR (1984). Factors associated with fresh meat color: a review. J. Food Qual..

[15] Attri P (2015). Influence of reactive species on the modification of biomolecules generated from the soft plasma. Sci. Rep..

[16] Renerre MT (1990). Factors involved in the discoloration of beef meat. Int. J. Food Sci. Technol..

[17] Traylor MJ (2011). Long-term antibacterial efficacy of air plasma-activated water. J. Phys. D: Appl. Phys..

[18] Jung S (2017). Direct infusion of nitrite into meat batter by atmospheric pressure plasma treatment. Innov. Food Sci. Emerg. Technol..

[19] Fox JB (1966). The chemistry of meat pigments. J. Agric. Food Chem..

[20] Lawrie R. A. *Lawrie’s meat science*. 7th ed. 280–290 (Cambridge, England: Woodhead, Ltd., 2006).

[21] Bondoc LL, Timkovich R (1989). Structural characterization of nitrimyoglobin. J. Biol. Chem..

[22] Fox JB, Thomson JS (1964). The formation of green heme pigments from metmyoglobin and methemoglobin by the action of nitrite. Biochemistry.

[23] Millar SJ, Moss BW, Stevenson MH (1996). Some observations on the absorption spectra of various myoglobin derivatives found in meat. Meat Sci..

[24] Nicol DJ, Shaw MK, Ledward DA (1970). Hydrogen sulfide production by bacteria and sulfmyoglobin formation in prepacked chilled beef. Appl. Environ. Microbiol..

[25] Hunt, M. C. *et al*. *AMSA meat color measurement guidelines*. 1–135 (Illinois, USA: American Meat Science Association, Champaign, 2012).

[26] Lee YW, Song KB (2002). Effect of γ-irradiation on the molecular properties of myoglobin. J. Biochem. Mol. Biol..

[27] Onuoha AC, Rusling JF (1995). Electroactive myoglobin-surfactant films in a bicontinuous microemulsion. Langmuir.

[28] Machala Z (2013). Formation of ROS and RNS in water electro‐sprayed through transient spark discharge in air and their bactericidal effects. Plasma Process. Polym..

[29] Oehmigen K (2011). Estimation of possible mechanisms of *Escherichia coli* inactivation by plasma treated sodium chloride solution. Plasma Process. Polym..

[30] Jung S (2015). The use of atmospheric pressure plasma-treated water as a source of nitrite for emulsion-type sausage. Meat Sci..

[31] Braida W, Ong SK (2000). Decomposition of nitrite under various pH and aeration conditions. Water Air Soil Pollut..

[32] Brisset JL, Pawlat J (2016). Chemical effects of air plasma species on aqueous solutes in direct and delayed exposure modes: discharge, post-discharge and plasma activated water. Plasma Chem. Plasma Process..

[33] Praneeth VKK, Neese F, Lehnert N (2005). Spin density distribution in five-and six-coordinate iron (II)−porphyrin NO complexes evidenced by magnetic circular dichroism spectroscopy. Inorg. Chem..

[34] Yi J, Richter-Addo GB (2012). Unveiling the three-dimensional structure of the green pigment of nitrite-cured meat. Chem. Commun..

[35] Fox JB, Thomson JS (1963). Formation of bovine nitrosylmyoglobin. I. pH 4.5–6.5. Biochemistry.

[36] Yong, H. I. *et al*. Color development, physiochemical properties, and microbiological safety of pork jerky processed with atmospheric pressure plasma. *Innov. Food Sci. Emerg. Technol*. In press, (2018).

