# Supplementary material for: Mechanism Underlying Green Discolouration of Myoglobin Induced by Atmospheric Pressure Plasma
Source: Sci Rep. 2018 Jun 28;8:9790. doi: 10.1038/s41598-018-28096-4 (PMC6023923; doi:10.1038/s41598-018-28096-4)
Supplement: Supplementary file 1 — Supplementary information [file 41598_2018_28096_MOESM1_ESM.docx]

**Supplementary Information**

**Mechanism Underlying Green Discolouration of Myoglobin Induced by Atmospheric Pressure Plasma**

Hae In Yong, Mookyoung Han, Hyun-Joo Kim, Jeong-Yong Suh, Cheorun Jo

**
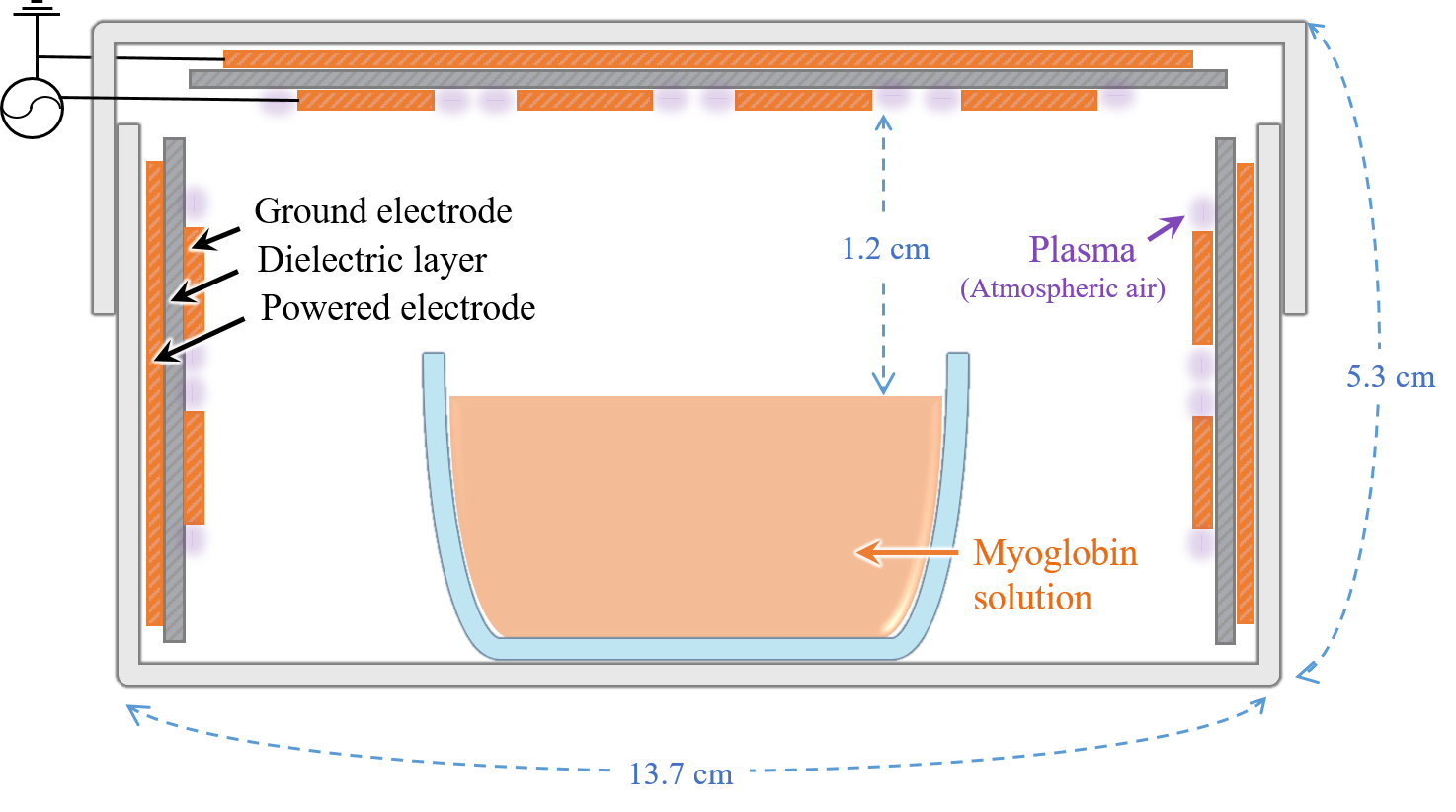
**

**Figure S1.** Detailed illustration of the atmospheric pressure plasma apparatus.

**
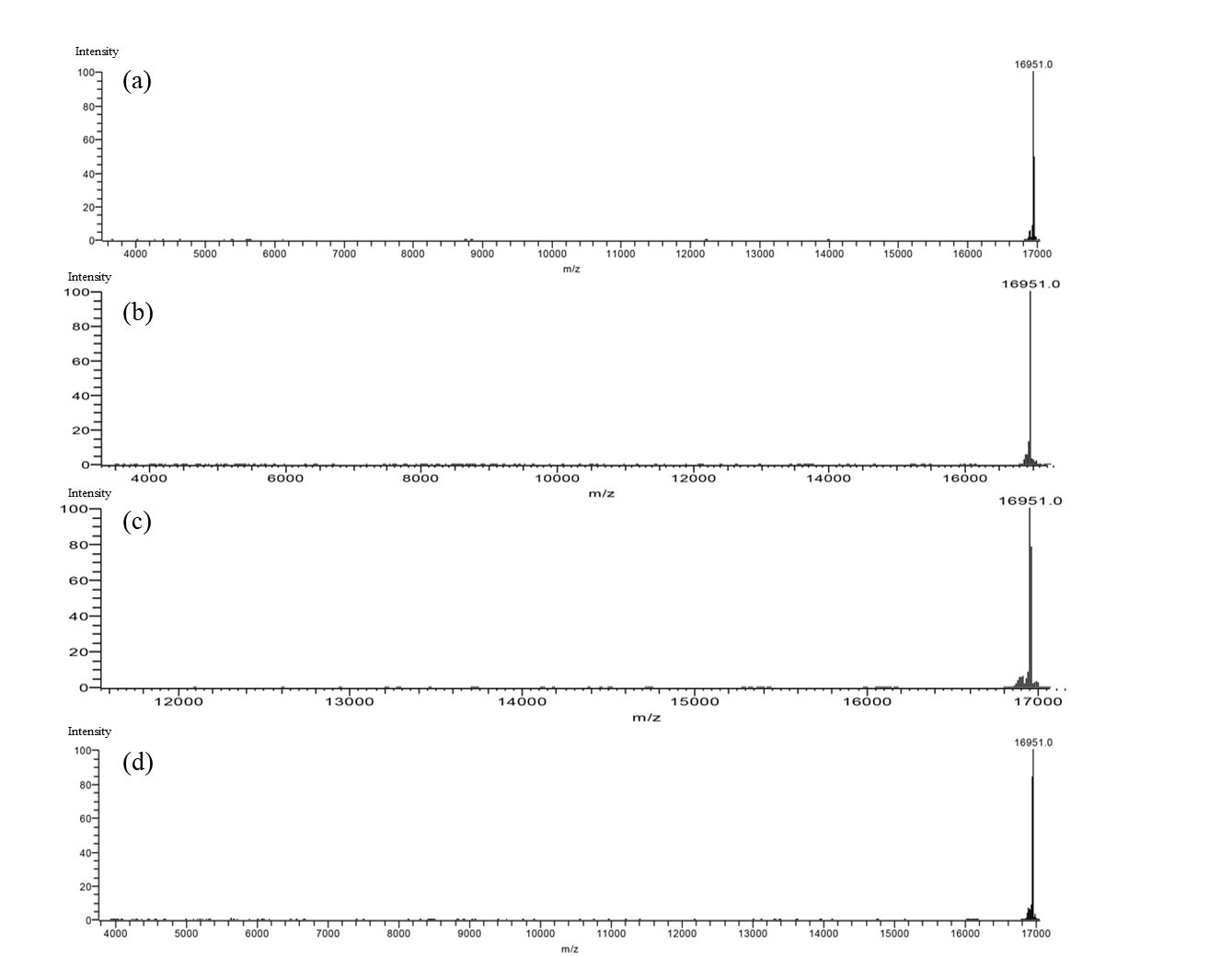
**

**Figure S2.** Electrospray ionization-mass spectra of myoglobin in phosphate buffer after the exposure of atmospheric pressure plasma for 0 (a), 5, (b), 10 (c), and 20 min (d).

**
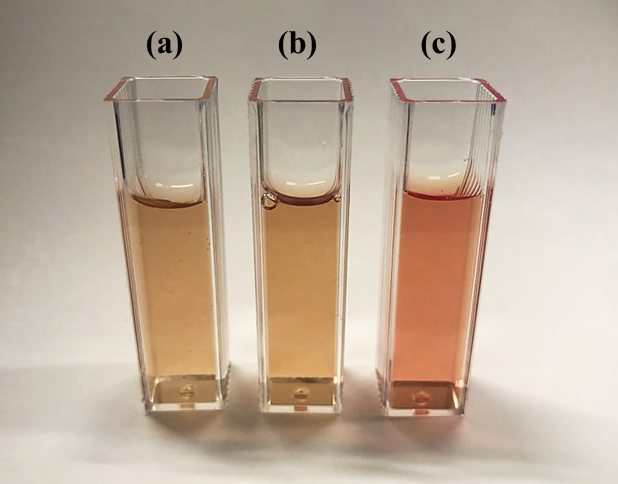
**

**Figure S3.** Visual appearance of myoglobin (a), and myoglobin supplemented with 0.1% (b) and 0.5% (c) sodium dithionite following exposure to atmospheric pressure plasma for 20 min. All myoglobin samples were dissolved in phosphate buffer.

**Table S1.** Color of protoporphyrin IX in phosphate buffer after the exposure of atmospheric pressure plasma

| Properties | Treatment time (min) | | | | SEM^1)^ |
| --- | --- | --- | --- | --- | --- |
|  | 0 | 5 | 10 | 20 |  |
| *L^*^* | 92.32^d^ | 93.47^c^ | 93.78^b^ | 93.92^a^ | 0.006 |
| *a^*^* | 0.80^a^ | 0.04^b^ | -0.03^c^ | -0.10^d^ | 0.003 |
| *b^*^* | 22.58^a^ | 19.45^b^ | 18.77^c^ | 18.55^c^ | 0.072 |
| Chroma | 22.59^a^ | 19.45^b^ | 18.77^c^ | 18.55^c^ | 0.073 |
| Δ*E* | 0.00^c^ | 3.41^b^ | 4.16^a^ | 4.42^a^ | 0.066 |

^1)^Standard error of the mean (n=12).

^a-d^Values with different letters within the same row differ significantly (*P*<0.05).
